# Enhancement of glycerol metabolism in the oleaginous marine diatom *Fistulifera solaris* JPCC DA0580 to improve triacylglycerol productivity

**DOI:** 10.1186/s13068-014-0184-9

**Published:** 2015-01-22

**Authors:** Masaki Muto, Masayoshi Tanaka, Yue Liang, Tomoko Yoshino, Mitsufumi Matsumoto, Tsuyoshi Tanaka

**Affiliations:** Division of Biotechnology and Life Science, Institute of Engineering, Tokyo University of Agriculture and Technology, 2-24-16 Naka-cho, Koganei, Tokyo 184-8588 Japan; JST, CREST, Sanbancho 5, Chiyoda-ku, Tokyo 102-0075 Japan; Biotechnology Laboratory, Electric Power Development Co., Ltd., Yanagisaki-machi, Wakamatsu-ku, Kitakyusyu 808-0111 Japan

**Keywords:** Oleaginous microalgae, *Fistulifera solaris*, Mixotrophic cultivation, Glycerol kinase, Biodiesel fuel, Metabolic engineering

## Abstract

**Background:**

Microalgal oil is a promising alternative feedstock for biodiesel fuel (BDF). Mixotrophic cultivation with glycerol, the primary byproduct of BDF production, may be used to optimize BDF production. This strategy would reduce costs through glycerol recycling and improve lipid productivity and biomass productivity by overcoming the growth retardation caused by decreased light penetration in high-density culture.

**Results:**

Overexpression of the endogenous glycerol kinase (*GK*) gene in an oleaginous marine diatom, *Fistulifera solaris* JPCC DA0580, accelerates glycerol metabolism and improves lipid and biomass productivities. Two candidates were selected from a collection of 90 G418-resistant clones, based on growth and confirmation of genome integration. *GK* gene expression was higher in the selected clones (GK1_7 and GK2_16) than in the wild-type culture. The GK2_16 clone achieved a 12% increase in lipid productivity.

**Conclusion:**

We have demonstrated the potential of metabolic engineering in oleaginous microalgae to improve lipid productivity. Metabolic engineering techniques can be used to optimize BDF production.

**Electronic supplementary material:**

The online version of this article (doi:10.1186/s13068-014-0184-9) contains supplementary material, which is available to authorized users.

## Introduction

The global energy crisis and climate change have aroused considerable interest in alternative fuels. Microalgae are widely accepted as one of the most promising biodiesel fuel (BDF) feedstocks because of their high photoautotrophic biomass productivity and neutral lipid content [1,2]. Various oleaginous microalgal strains, including *Nannochloropsis* sp., *Botryococcus braunii*, and *Chlorella sorokiniana,* are promising candidates for BDF production [2-4]. A transformable [5] oleaginous marine pennate diatom, *Fistulifera solaris* JPCC DA0580, was the strongest producer of triacylglycerol (TAG) in a collection of 1,393 strains of domestic marine microalgae [6,7]. The lipid content of *F. solaris* is comparable to that of *Nannochloropsis* sp. [2,8]. The fatty acid composition of *F. solaris* is also similar to that of *Nannochloropsis* sp., with high lipid quality for BDF production, as demonstrated by cetane number, kinematic viscosity, iodine value, and oxidative stability [9]. A high density cultivation technique and outdoor cultivation have been used for *F. solaris* [8,10]. The whole genome of *F. solaris* has been sequenced, and the chloroplast genome has been published [11]. This knowledge has empowered further study to enhance BDF productivity and quality in *F. solaris*. However, industrial application of microalgal BDF is hampered by a combination of high cost and low energy yield. One strategy to overcome these problems is to utilize the byproducts of BDF production.

Recycling of glycerol, the major byproduct of BDF production, may help reduce the cost of BDF [12,13]. Indeed, production of BDF from 1 kg of microalgal TAG releases approximately 0.1 kg of crude glycerol [12]; such yields could decrease the global price of glycerol. Mixotrophic cultivation of various microalgae enhances biomass productivity as well as the production of corresponding valuable bioproducts such as eicosapentaenoic acid (EPA) [14], lipids for BDF [4,15], and pigments [14,16]. Overexpression of glycerol kinase (GK) in the transformed yeast *Saccharomyces cerevisiae* improves lipid productivity [17], since the first reaction of glycerol metabolism occurs when GK converts glycerol to glycerol-3-phosphate (G3P), a precursor for TAG biosynthesis through the Kennedy pathway [18]. Overexpression of the *GK* gene leads to accumulation of intracellular G3P, which might then stimulate TAG synthesis and accumulation. Genetic engineering of GK to improve glycerol metabolism and lipid productivity has not been performed in microalgae.

Metabolic engineering to enhance microalgal BDF production has only been achieved in model microalgae such as the green alga *Chlamydomonas reinhardtii* [19] and the diatoms *Thalassiosira pseudonana* [20] and *Phaeodactylum tricornutum* [21,22]. However, *C. reinhardtii*, *T. pseudonana*, and *P. tricornutum* are not ideal for BDF production because their lipid content is moderate [23]. In the oleaginous microalgae, stable transformation has only been established for *Nannochloropsis* sp. [2,24] and *F. solaris* [5], neither of which has been metabolically engineered.

In this study, *F. solaris* was transformed with a vector containing the endogenous *GK* gene. Glycerol utility, biomass productivity, and lipid productivity were higher than in the wild-type strain. Overexpression of the *GK* gene in the marine diatom *F. solaris* may have great utility in commercial BDF production.

## Results and discussion

### Identification of glycerol kinase genes in *Fistulifera solaris*

Two endogenous *GK* genes in *F. solaris*, designated *GK1* [DDBJ/EMBL/GenBank:LC009547 (Gene ID: g11728)] and *GK2* [DDBJ/EMBL/GenBank:LC009548 (Gene ID: g14921)], were identified in the draft genome sequence. The nucleotide and amino acid sequences of *GK1* and *GK2* exhibited 92% nucleotide sequence similarity and 93% amino acid sequence similarity. Both genes are identical in length (1827 bp) and are relatively longer than the glycerol kinase genes (1728 bp and 1737 bp) previously identified in the model diatom *Thalassiosira pseudonana* [GenBank IDs: XP_002296792 and XP_002295903]. *GK1* in *F. solaris* shares 58% sequence identity and *GK2* shares 57% sequence identity with the *GK* [XP_002296792] of *T. pseudonana*. The amino acid sequences of GK1 and GK2 in *F. solaris* and the GKs in *T. pseudonana* contain an FGGY carbohydrate kinase domain, characteristic of glycerol kinases [25].

### Selection of candidate clones based on cell growth

**Figure 1:**
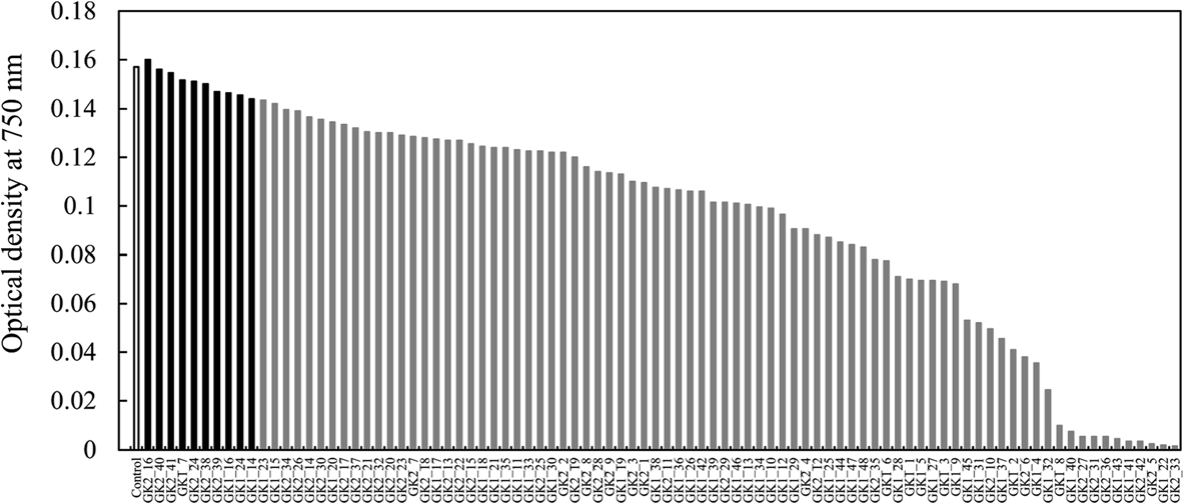
**Final cell densities of the transformants (optical density at 750 nm).** The glycerol kinase (*GK*) transformants were generated with pSP-GK1/fcpB or pSP-GK2/fcpB. The white bar indicates the cell density of the negative control transformed with pSP-NPT/H4 (n = 5). The black bar indicates 10 clones with cell densities similar to that of the negative control.

After transformation with pSP-GK1 and pSP-GK2, 48 *GK1* clones (GK1_1 to GK1_48) and 42 *GK2* clones (GK2_1 to GK2_42) were obtained from an agar plate containing the antibiotic reagent G418. These clones were cultured in 96-well microtiter plates, and the final cell density was evaluated by determining the optical density to estimate the effect of genetic transformation on cell growth. The final cell densities of the transformants varied from 1% to 102% of the mean density of the control strains (n = 5), which were transformed with the empty pSP-NPT/H4 vector (Figure 1). The variation of transformant cell density may be derived from different loci and copy numbers of the integrated transgenic fragments [5,26,27]. The final cell densities of 10 pSP-GK1 and pSP-GK2 transformants, including GK2_16, GK2_40, GK2_41, GK1_7, GK2_24, GK2_38, GK2_39, GK1_16, GK1_24, and GK1_14 (Figure 1, black columns), were not significantly varied as compared with the mean ± SD (OD = 0.157 ± 0.015) of the control strains (n = 5), and were selected for subsequent experiments.

### Confirmation of the integrated *GK* genes from the transformants

**Figure 2:**
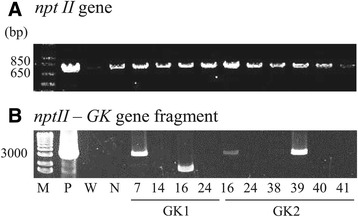
**Heterologous gene detection in** ***Fistulifera solaris*** **transformed with pSP-GK1/fcpB or pSP-GK2/fcpB.** The *nptII* **(A)** and *nptII*-glycerol kinase (*GK*) gene fragments **(B)** were PCR-amplified. M, 1-kb marker; P, pSP-GK1/fcpB as the positive control; W, wild type; N, negative control transformed with pSP-NPT/H4; GK1, transformed with pSP-GK1/fcpB; GK2, transformed with pSP-GK2/fcpB.

The presence of the integrated genes in the 10 selected clones was verified by PCR (Figure 2). A 795-bp fragment encoding the *nptII* gene region was amplified from the genomic DNA of G418-resistant clones. All 10 clones, as well as the negative control with the empty pSP-NPT/H4 vector, showed the presence of the *nptII* gene, while no amplified product was obtained from the wild type. On the other hand, the combined fragment of the *nptII* gene and the *GK1 or GK2* gene region (2622 bp) was amplified from only three of the clones (GK1_7, GK2_16, and GK2_39). The wild type and the negative control showed no *nptII*-*GK* product. These results suggest that partial gene integration of the vector may occur during transformation in *F. solaris*. The GK2_39 clone showed a decrease in growth rate (Additional file 1) and was eliminated from subsequent experiments.

**Figure 3:**
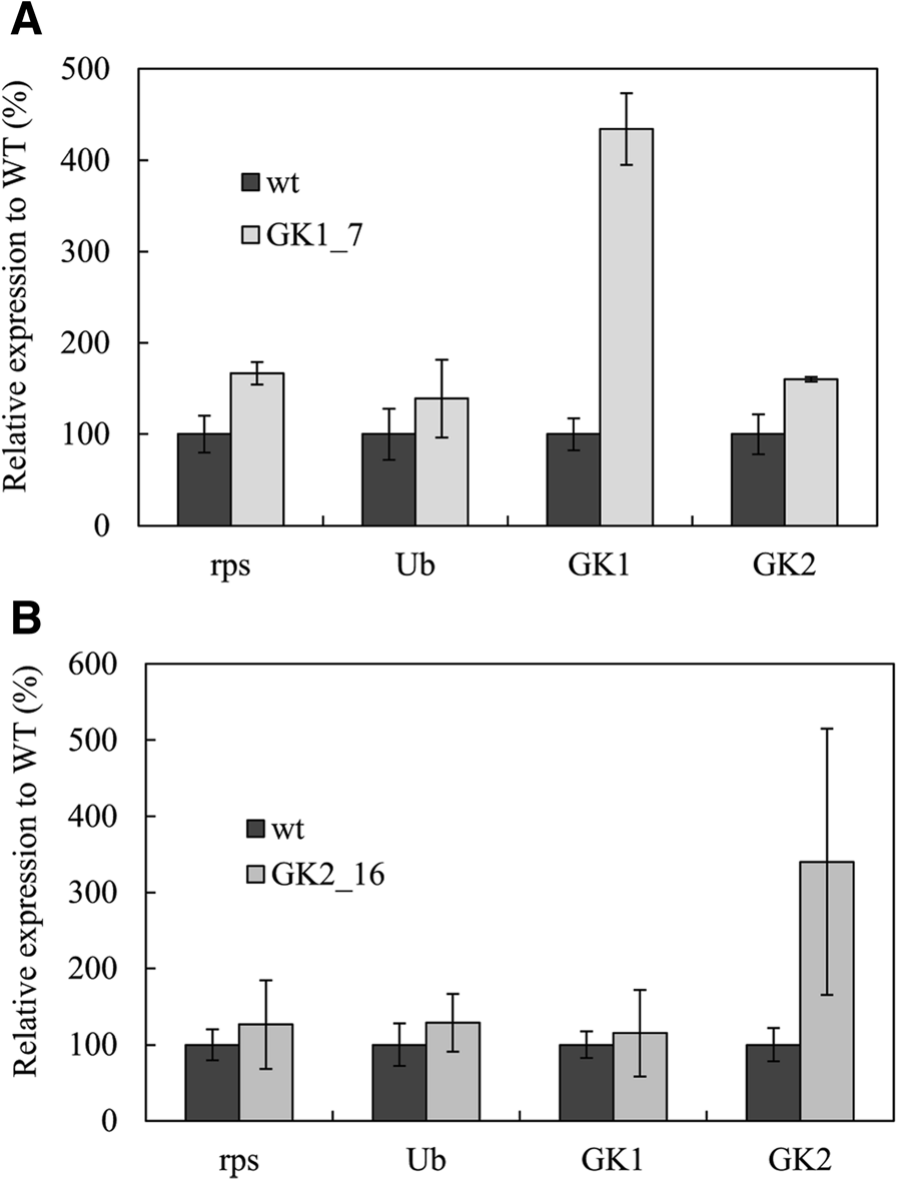
**Relative mRNA abundance in the wild-type and glycerol kinase (** ***GK*** **) gene transformants.** Gene expression was evaluated in the GK1_7 **(A)** and GK2_16 **(B)** transformants, with housekeeping genes *rps* and *Ub* as controls.

*GK* expression levels in GK1_7 and GK2_16 clones were examined by quantitative reverse transcription PCR (qRT-PCR), with housekeeping genes *ribosomal protein subunit* (*rps*) and ubiquitin (*Ub*) as controls (Figure 3). The *rps* and *Ub* genes showed similar expression levels in the transformants and wild-type strains. In contrast, large differences were observed in *GK1* and *GK2* gene expression. *GK1* expression in the GK1_7 clone increased by 4.3-fold in comparison to that observed in the wild type (Figure 3A) and *GK2* expression in the GK2_16 clone increased by 3.4-fold (Figure 3B). These results indicate that *GK* gene expression was enhanced in the GK1_7 and GK2_16 transformants. Moreover, expression of the *GK2* gene in the GK1_7 clone and of the *GK1* gene in the GK2_16 clone changed negligibly, suggesting that specific overexpression of *GK1* and *GK2* had been achieved. However, based on the comparison of the cycle threshold (Ct) values, the expression levels of *GK* (Ct = 22.5) seemed to be lower than those of the two housekeeping genes *rps* (Ct = 20.6) and *Ub* (Ct = 21.6). Further enhancement of the *GK* gene expression would be attained in the future through the optimization of the promoter region.

### Evaluation of lipid productivity in transformants

Prior to exploring glycerol utilization by transformants, we investigated the optimum glycerol concentration for mixotrophic growth in wild-type *F. solaris* (Additional file 2). The highest cell density was obtained when the initial glycerol concentration was 50 mM, similar to reports in other microalgae such as the marine diatom *P. tricornutum* (100 mM) and the green alga *Chlorella vulgaris* (110 mM) [14,15]. Growth was inhibited at more than 250 mM glycerol, perhaps because of the increasing viscosity of the culture media. Based on these results, we chose to use 50 mM glycerol for mixotrophic growth.

To investigate lipid productivity, the final cell concentration, dry cell weight, lipid content, and lipid yield were evaluated in the transformants and wild-type strains. Table 1 summarizes the data for transformants and wild-type cultures in the absence of glycerol. The GK1_7 clones had a significantly lower lipid content in comparison to the wild type and GK2_16, leading to a slightly lower lipid yield in GK1_7, although the final cell concentration and dry cell weight of this strain were increased. In comparison to the wild type, GK2_16 showed a slightly increased dry cell weight and lipid content, thus increasing the final lipid yield. The GK2_16 results suggest that, even under photoautotrophic culture conditions, *GK* overexpression may also affect glycerol utilization and result in variable cell growth and lipid production.

**Table 1:** **Biomass and lipid productivities in transformants cultivated in f/2 medium**

**Clone**	**Final cell density**	**Dry cell weight**	**Lipid content**	**Lipid yield**
	**(× 10** ^**7**^ **cells/mL)**	**(mg/L)**	**(% dry w/w)**	**(mg/L)**
Wild type	1.03 ± 0.04	407 ± 12	36.2 ± 0.8	148 ± 7
GK1_7	1.12 ± 0.03	426 ± 11	31.7 ± 3.5	135 ± 15
GK2_16	1.03 ± 0.08	426 ± 49	40.9 ± 1.3	152 ± 13

Table 2 summarizes the data of the wild type and the transformants cultured with 50 mM glycerol. The increases in final cell concentration, dry cell weight, lipid content, and lipid yield were compared in wild-type cultures with and without glycerol (Table 1). This result suggests that it is possible to improve BDF production by mixotrophic cultivation of *F. solaris*. As expected, increases in final cell concentration (GK1_7: 108%, GK2_16: 116%) and dry cell weight (GK1_7: 103%, GK2_16: 110%) were observed in both transformants; however, in comparison to GK1_7 cultured without glycerol (Table 1), the addition of glycerol did not significantly affect all tested parameters, suggesting that the *GK*1 gene may not be fully functional. The *GK1* gene in GK1_7 may be integrated into unexpected loci in the genome, thus affecting the lipid content in this strain. On the other hand, all of the tested parameters significantly increased in the GK2_16 clone versus the same clone cultured without glycerol, and a 112% increase in lipid yield was observed in comparison to that observed in the wild type. These results suggest that GK2 is fully functional in this transformant. Remaining glycerol concentrations in the filtered culture media of the GK2_16 clone were measured by HPLC. Glycerol consumption was markedly increased to 2.4 mM (221.0 mg/L) in the GK2_16 clone in comparison to the wild type (1.7 mM, 156.6 mg/L) cultivated in f/2 medium containing 7.5 mM glycerol. Overexpression of the *GK2* gene resulted in a 1.4-fold increase in glycerol utilization, which is the most likely explanation for the increase in biomass and lipid productivity.

**Table 2:** **Biomass and lipid productivities in transformants cultivated with 50 mM glycerol**

**Clone**	**Final cell density**	**Dry cell weight**	**Lipid content**	**Lipid yield**
	**(× 10** ^**7**^ **cells/mL)**	**(mg/L)**	**(% dry w/w)**	**(mg/L)**
Wild type	1.06 ± 0.03	432 ± 12	38.6 ± 2.8	167 ± 12
GK1_7	1.14 ± 0.07	443 ± 30	30.3 ± 0.6	134 ± 6
GK2_16	1.23 ± 0.10	477 ± 56	39.6 ± 4.3	187 ± 11

Glycerol can be utilized as the sole carbon source in various pathways including glucose metabolism and CO_2_ fixation. A GK mutant of *Arabidopsis* which was not able to utilize glycerol showed no variance in lipid content [28]. This study suggested that GK in essential for glycerol metabolism; however, glycerol metabolism is not directly linked to lipid synthesis. On the other hand, by increasing the G3P level through the enhancement of GK activity by metabolic engineering, TAG accumulation was reported to be induced in higher plants and yeast [17,29]. Thus, we propose to overexpress *GK* to clarify whether the glycerol metabolism in *F. solaris* is linked with TAG synthesis.

## Conclusion

The lipid productivity of microalgae closely depends on the basic physical laws of photosynthesis. Mixotrophic cultivation could overcome the mutual shading of cells under high cell density photoautotrophic cultivation. In other words, mixotrophic cultivation may reduce the dependence of cell growth on the supply of sunlight for photosynthesis in large-scale cultivation. By enhancing organic carbon assimilation through metabolic engineering, the oleaginous microalgae may become a reliable, renewable feedstock for BDF production.

In this study, the *GK* gene was overexpressed in the oleaginous microalga *F. solaris* to accelerate glycerol metabolism. The transformant GK2_16 showed a 12% increase in lipid productivity. To our knowledge, this is the first report of metabolic engineering in oleaginous microalgae that has focused on enhanced lipid productivity. Our results and observations will promote the development of technologies to improve lipid productivity through glycerol recycling. The establishment of metabolic engineering techniques for the oleaginous microalgae has great potential to optimize BDF production. Through further metabolic engineering of this oleaginous microalga, improved BDF quality and productivity can be expected.

## Materials and methods

### Strain and culture conditions

The marine pennate diatom *F. solaris* JPCC DA0580 was isolated from the mouths of the Sumiyo and Yakugachi Rivers in Amami-Ohshima, Kagoshima, Japan [7,30]. The cells were cultured as previously reported [5]. Briefly, pre-cultivated cells containing approximately 40% lipid component were inoculated in half-strength Guillard’s ”f” medium (f/2) [31] at an initial density of 1 × 10^5^ cells/mL. The cells were grown at 25°C under continuous, cool-white fluorescent lights (140 μmol/m^2^/s). The cell density was estimated using a hemocytometer.

For the purpose of positive transformant selection, G418-resistant colonies were cultured with f/2 liquid medium containing 500 μg/mL G418 (Roche Applied Science, Indianapolis, IN), an aminoglycoside antibiotic, in a 96-well plate (Sumilon, Tokyo, Japan) at 25°C under constant illumination of 50 μmol/m^2^/s. After three weeks, 20 μL of the G418-resistant clone cultures were transferred to a fresh 96-well plate and incubated for another six days. In order to determine the cell density, the optical density (at 750 nm) of each well was measured in a microplate reader (SH-9000, Corona Electric, Ibaraki, Japan). Furthermore, to evaluate the effect of glycerol on cell growth, wild-type and transformant strains were cultured in the presence of 10 mM to 500 mM glycerol.

### Plasmid construction

A homology search, using BLASTX, was performed with reference to the 19,859 genes from the draft genome sequence of *F. solaris* to obtain the GK-coding genes [11] (the whole genome is undergoing preparation for publication). The pSP-NPT/H4 vector [5] was used to construct the expression vector for the two predicted *GK* genes. The nucleotide sequence comprising a promoter region (500 bp) from a fucoxanthin chlorophyll *a/c*-binding protein B (*fcpB*) gene in *F. solaris* and a terminator region (227 bp) from a fucoxanthin chlorophyll a/c-binding protein A (*fcpA*) gene [27] in *P. tricornutum* was synthesized (Integrated DNA Technologies, Inc., Coralville, IA, USA) and cloned into the *Sal*I-*Sph*I site of pSP-NPT/H4. The *GK1* and *GK2* gene fragments, with restriction sites (*Eco*RI and *Pst*I), were PCR-amplified from *F. solaris* genomic DNA. These fragments were then inserted into the *Eco*RI-*Pst*I site within the expression vector pSP-NPT/H4 to construct pSP-GK1 and pSP-GK2 (Additional file 3). Transformants carrying the empty pSP-NPT/H4 vector served as the negative control.

### Transformation

The transformation of *F. solaris* was performed by microparticle bombardment, as described (Biolistic PDS-1000/He Particle Delivery System; Bio-Rad Laboratories, Inc., Hercules, CA) [5]. Tungsten particles (3 mg; 0.6 μm in diameter; Kojundo Chemical Laboratory, Saitama, Japan) were coated with pSP-GK1 or pSP-GK2 (5 μg), according to the manufacturer’s protocols. Microalgal cells were harvested at mid-log phase. Approximately 5 × 10^7^ cells in the agar plate were bombarded with 600 ng plasmid DNA by using an 1,100-psi rupture disk under a negative pressure of 28 in of Hg. After bombardment, the cells were incubated on plates at 25°C under constant illumination (140 μmol/m^2^/s^1^) for 24 h and then resuspended in 1 mL f/2 medium. Then, approximately 10^7^ cells were inoculated at 25°C under constant illumination on a 1% f/2 plate containing 500 μg/mL G418. G418-resistant colonies were obtained after three weeks.

### PCR and qRT-PCR

Successful integration of the target gene region, including the promoter, *GK* gene, and terminator, was confirmed by PCR. Selected G418-resistant clones (approximately 4 × 10^6^ cells) were used for direct PCR. The full-length *nptII* gene (795 bp), *GK* genes (1.9 kb), and *nptII-GK* gene fragment (3.2 kb) were PCR-amplified with specific primer sets (Additional file 4) and TaKaRa Taq polymerase (TaKaRa Bio, Otsu, Japan) under the following cycling conditions: 94°C for 1 min, (94°C for 30 s, 60°C for 30 s, and 72°C for 2 min) × 30 cycles, and 72°C for 5 min. Amplification products were analyzed by electrophoresis.

qRT-PCR was performed to measure *GK* expression in selected transformants. Total RNA was isolated with Plant RNA Isolation Reagent (Invitrogen Corporation) according to manufacturer instructions. The extracts were further purified with the RNeasy Mini kit (Qiagen, Valencia, CA). After purification, the total RNA solution was treated with DNase I (TaKaRa). The cDNA was synthesized by using a PrimeScript II First Strand cDNA Synthesis Kit (TaKaRa) and 1 μg total RNA. The cDNAs were PCR-amplified to determine the transcript levels of *GK1* and *GK2*. The housekeeping genes *rps* and *Ub* were used as controls. Glyceraldehyde-3-phosphate dehydrogenase (GAPDH) was used for standardization between samples [32]. Amplified fragments were purified with a QIAquick PCR Purification Kit (Qiagen). After DNA concentrations were measured by e-spect UV-Vis spectrometry (Malcom, Tokyo, Japan), the purified DNA fragments were diluted and used to obtain standard curves. Individual qRT-PCR reactions contained 10 μL Fast SYBR Green Master Mix (Applied Biosystems), 2 μL of 1 ng/μL cDNA, and 1 μL of 10 mM forward and reverse primers. Two-step qRT-PCR amplification (40 cycles of 95°C for 3 s, followed by 60°C for 30 s) was performed on a ViiA 7 Real-Time PCR System (Life Technologies). Based on the standard curves, the quantities of *GK1*, *GK2*, *rps*, and *Ub* transcripts were calculated relative to the wild-type sample. The data represented an average of three independent biological samples.

### Cell lipid content and glycerol concentration in the medium

Lipids were extracted from lyophilized microalgal cells (about 50 mg) with 10 mL *n*-hexane as described [30]. The crude extracts were dried under argon gas, and the ratio of dried lipid weight to dried cell weight was defined as the lipid content.

To determine glycerol consumption in the wild-type and transformant strains, cells were cultured in f/2 medium containing 7.5 mM glycerol. This low concentration enabled us to detect significant variations in glycerol consumption. The glycerol concentration of the filtered culture supernatants was measured on a Shimadzu Prominence HPLC system (Shimadzu Scientific Instruments, Kyoto, Japan) equipped with an Aminex HPX-87H+ column (300 mm × 7.8 mm, Bio-Rad Laboratories). The column was maintained at 50°C. Glycerol was eluted with 25 mM NH_4_HCO_2_ at 1.0 mL/min and detected with an Evaporative Light Scattering Detector System (Shimadzu).

## Additional files

Additional file 1:
**Growth curves of the wild-type (solid triangles) and glycerol kinase (** ***GK*** **)-expressing lines GK1_7 (open circles), GK2_16 (large open squares), and GK2_39 (small open diamonds) during flat flask cultivation in f/2 medium.**
Additional file 2:
**Final cell density of the wild-type (gray bar) and glycerol kinase (** ***GK*** **) gene transformant (GK2_16; white bar) in the presence of glycerol.**
Additional file 3:
**Restriction map of the pSP-GK/fcpB (pSP-GK1/fcpB, pSP-GK2/fcpB) vector for** ***GK*** **overexpression.** Abbreviations: H4 promoter, histone H4 gene promoter from *Fistulifera solaris*; *nptII* gene, neomycin phosphotransferase gene; Terminator, fucoxanthin chlorophyll *a/c*-binding protein A gene terminator from *Phaeodactylum tricornutum*; *fcpB* promoter, fucoxanthin chlorophyll *a/c*-binding protein B gene promoter from *F. solaris*; glycerol kinase gene, glycerol kinase gene (g10050, g13546) from *F. solaris*. Additional file 4:
**Primers used for quantitative qRT-PCR and PCR to obtain the glycerol kinase (** ***GK*** **) overexpression clone.**

## Abbreviations

BDF: biodiesel fuel
BLAST: Basic Local Alignment Search Tool
bp: base pairs
Ct: cycle threshold
*fcpA*: fucoxanthin chlorophyll *a/c*-binding protein A
*fcpB*: Fucoxanthin chlorophyll *a/c*-binding protein B
G3P: glycerol-3-phosphate
GAPDH: glyceraldehyde-3-phosphate dehydrogenase
GK: glycerol kinase
HPLC: high performance liquid chromatography
PCR: polymerase chain reaction
qRT-PCR: quantitative reverse transcription PCR
*rps*: ribosomal protein subunit
TAG: triacylglycerol
*Ub*: ubiquitin
