# Supplementary figures and images for: Enhancement of glycerol metabolism in the oleaginous marine diatom Fistulifera solaris JPCC DA0580 to improve triacylglycerol productivity
Source: Biotechnol Biofuels. 2015 Jan 22;8:4. doi: 10.1186/s13068-014-0184-9 (PMC4308894; doi:10.1186/s13068-014-0184-9)

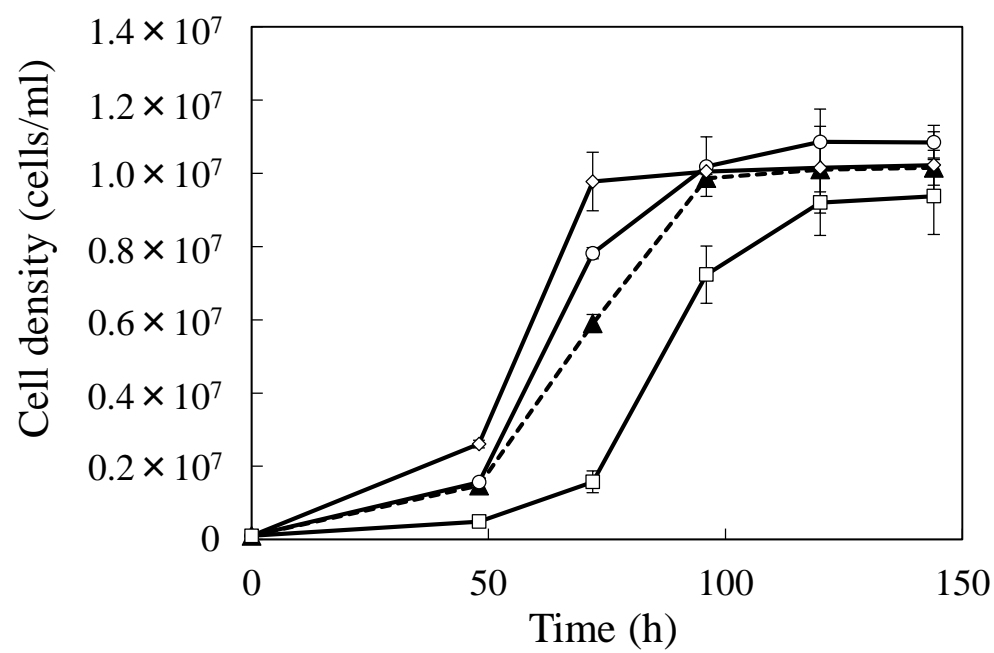

Supplement: Additional file 1: — Growth curves of the wild-type (solid triangles) and glycerol kinase ( GK )-expressing lines GK1_7 (open circles), GK2_16 (large open squares), and GK2_39 (small open diamonds) during flat flask cultivation in f/2 medium. [file 13068_2014_184_MOESM1_ESM.pdf]

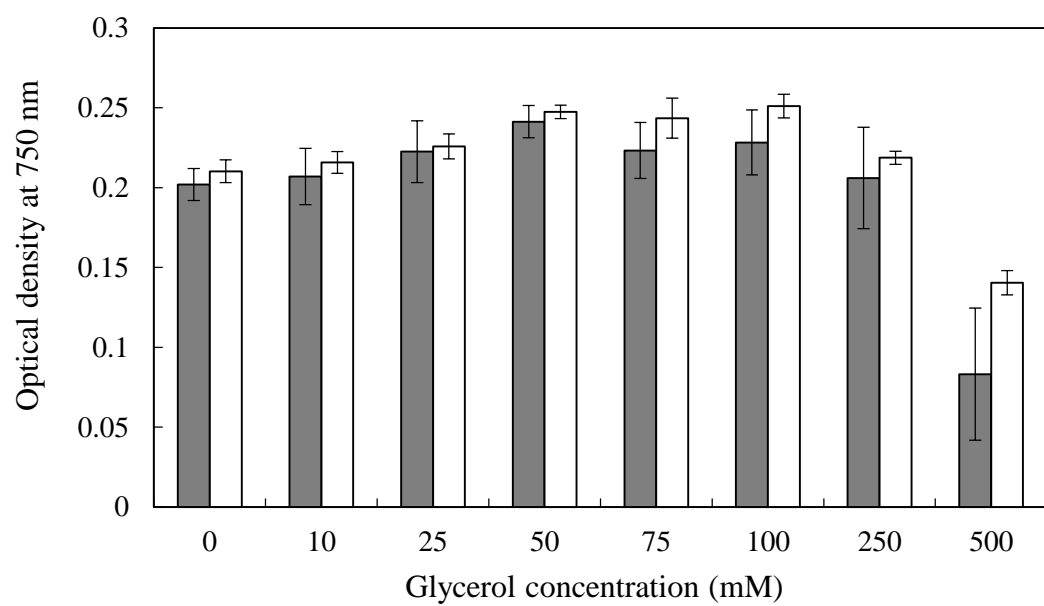

Supplement: Additional file 2: — Final cell density of the wild-type (gray bar) and glycerol kinase ( GK ) gene transformant (GK2_16; white bar) in the presence of glycerol. [file 13068_2014_184_MOESM2_ESM.pdf]

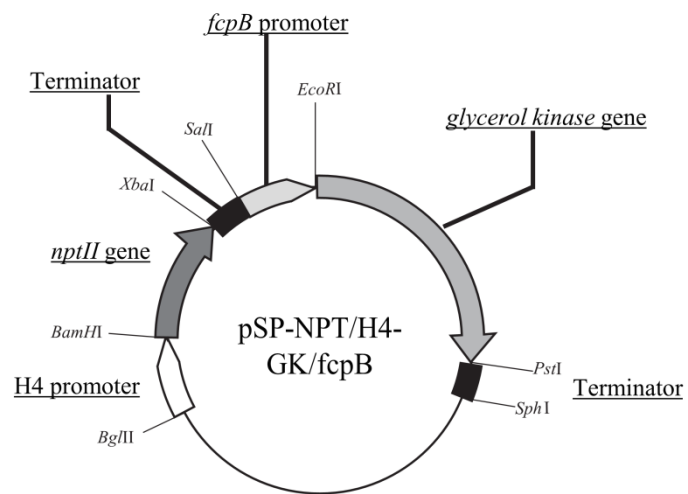

Supplement: Additional file 3: — Restriction map of the pSP-GK/fcpB (pSP-GK1/fcpB, pSP-GK2/fcpB) vector for GK overexpression. Abbreviations: H4 promoter, histone H4 gene promoter from Fistulifera solaris; nptII gene, neomycin phosphotransferase gene; Terminator, fucoxanthin chlorophyll a/c-binding protein A gene terminator from Phaeodactylum tricornutum; fcpB promoter, fucoxanthin chlorophyll a/c-binding protein B gene promoter from F. solaris; glycerol kinase gene, glycerol kinase gene (g10050, g13546) from F. solaris. [file 13068_2014_184_MOESM3_ESM.pdf]
