# Supplementary material for: Enhancement of glycerol metabolism in the oleaginous marine diatom Fistulifera solaris JPCC DA0580 to improve triacylglycerol productivity
Source: Biotechnol Biofuels. 2015 Jan 22;8:4. doi: 10.1186/s13068-014-0184-9 (PMC4308894; doi:10.1186/s13068-014-0184-9)
Supplement: Additional file 4: — Primers used for quantitative qRT-PCR and PCR to obtain the glycerol kinase ( GK ) overexpression clone. [file 13068_2014_184_MOESM4_ESM.pdf]

Gene	Accession No.	Primer name	Sequence	Amplicon size (bp)
**Used for PCR detection**				
nptII	-	nptIIFw	ATGATTGAACAAGATGGATTGC	795
		nptIIRv	TCAGAAGAACTCGTCAAGAAGG	
glycerol kinase	XXX, XXX	GKFw	ATTTGACTGCTACAGAATTCATGC	1900
		GKRv	TTGTTCTGCAGTTAGTTTAAGCG	
**Used for Real-time PCR**				
glycerol kinase	XXXX	g11728Fw	AGCTACTTCGCTGGGACAAA	127
		g11728Rv	GAAAGACGAGCCAAGAATCG	
	XXX	g14921Fw	AACGACCCCTGACAATGAAG	108
		g14921Rv	GAGGCTGTCATCCCAACAAT	
GAPDH	XXXX	g19411Fw	ATTGGAGTCAATGGCTTTGG	147
		g19411Rv	GCCATGAACGGAATCGTACT	
rps	XXXX	g2459Fw	CAAGAGAACGGCAAATTGGT	108
		g2459Rv	TTTGAGGGCCTTGACGATAC	
Ub	XXXX	g19562Fw	GCCGGTAAACAATTGGAAGA	146
		g19562Rv	GCCTTGTCGCAGTTGAATTT	
